# A Spontaneous In Situ Thiol-Ene Crosslinking Hydrogel with Thermo-Responsive Mechanical Properties

**DOI:** 10.3390/polym16091264

**Published:** 2024-05-01

**Authors:** Andreas Aerts, Maxim Vovchenko, Seyed Ali Elahi, Rocío Castro Viñuelas, Tess De Maeseneer, Martin Purino, Richard Hoogenboom, Hans Van Oosterwyck, Ilse Jonkers, Ruth Cardinaels, Mario Smet

**Affiliations:** 1 Laboratory of Organic Material Synthesis, Polymer Chemistry and Materials, Department of Chemistry, KU Leuven, Celestijnenlaan 200f, P.O. Box 2404, 3001 Leuven, Belgium; andreas.aerts@kuleuven.be; 2 Biomechanics Section, Department of Mechanical Engineering, KU Leuven, Celestijnenlaan 300C, P.O. Box 2419, 3001 Leuven, Belgium; 3 Laboratory for Soft Matter and Biophysics, Department of Physics and Astronomy, KU Leuven, Celestijnenlaan 200D, P.O. Box 2416, 3001 Leuven, Belgium; 4 Human Movement Biomechanics Research Group, Department of Movement Sciences, KU Leuven Tervuursevest 101, P.O. Box 1501, 3001 Leuven, Belgium; 5 Laboratory for Tissue Homeostasis and Disease, Department of Development and Regeneration, KU Leuven, Herestraat 49, P.O. Box 813, 3000 Leuven, Belgium; 6 Rheology and Technology, Soft Matter, Department of Chemical Engineering, KU Leuven, Celestijnenlaan 200J, P.O. Box 2424, 3001 Leuven, Belgium; 7 Supramolecular Chemistry Group, Department of Organic and Macromolecular Chemistry, UGent, Krijgslaan 281, Building S4, 9000 Ghent, Belgium; 8 Prometheus, Division of Skeletal Tissue Engineering, KU Leuven, Herestraat 49, P.O. Box 813, 3000 Leuven, Belgium

**Keywords:** hydrogel, Michael-type addition, N-isopropylacrylamide

## Abstract

The thermo-responsive behavior of Poly(N-isopropylacrylamide) makes it an ideal candidate to easily embed cells and allows the polymer mixture to be injected. However, P(NiPAAm) hydrogels possess minor mechanical properties. To increase the mechanical properties, a covalent bond is introduced into the P(NIPAAm) network through a biocompatible thiol-ene click-reaction by mixing two polymer solutions. Co-polymers with variable thiol or acrylate groups to thermo-responsive co-monomer ratios, ranging from 1% to 10%, were synthesized. Precise control of the crosslink density allowed customization of the hydrogel’s mechanical properties to match different tissue stiffness levels. Increasing the temperature of the hydrogel above its transition temperature of 31 °C induced the formation of additional physical interactions. These additional interactions both further increased the stiffness of the material and impacted its relaxation behavior. The developed optimized hydrogels reach stiffnesses more than ten times higher compared to the state of the art using similar polymers. Furthermore, when adding cells to the precursor polymer solutions, homogeneous thermo-responsive hydrogels with good cell viability were created upon mixing. In future work, the influence of the mechanical micro-environment on the cell’s behavior can be studied in vitro in a continuous manner by changing the incubation temperature.

## 1. Introduction

Hydrogels consist of natural or synthetic polymers cross-linked into a network [1]. The hydrophilicity of the polymers renders them capable of holding a huge amount of water while being resistant to dissolution due to the cross-linked nature of the network. In the past decades, hydrogels are increasingly being deployed as cell culturing environments given their favorable water content, permeability, and mechanical properties similar to that of soft tissue. As such, they optimally mimic salient elements found in the native extracellular matrix (ECM) [2]. Furthermore, hydrogels can be functionalized to make them responsive to one or more stimuli in their environment, such as pH [3], temperature [4], ionic strength [5], electric field [6], molecular recognition [7], and light [8,9,10,11,12,13,14]. Among the different possible stimuli, temperature is one of the most exploited ones because of its high applicability, non-contact functionality, controllability, and reliability [15,16,17,18,19]. Thermo-responsive hydrogels consist of temperature-sensitive polymers of which the solubility balance is dependent on temperature, causing a structural and mechanical change of the hydrogel network as a function of temperature. The most commonly studied thermo-responsive polymers are polymers with lower critical solution temperature (LCST) behavior, which implies that they undergo a solubility phase transition from a soluble one-phase system to a phase-separated, two-phase system upon heating [16]. The most common polymer with LCST behavior in water is poly(N-isopropylacrylamide), P(NiPAAm), and its co-polymers. The LCST phase transition temperature of P(NiPAAm), termed cloud point temperate (T_cp_) if recorded at a single concentration, is around physiological temperature (~32 °C) [20] and a good biocompatibility has been reported [21].

**Figure 1:**
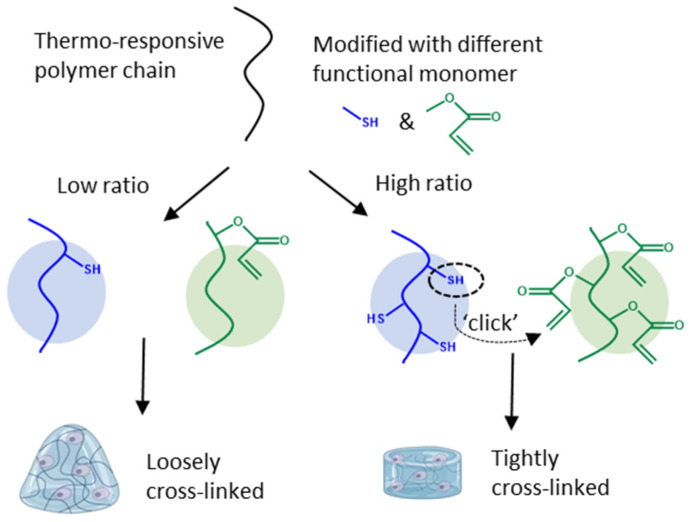
Graphical representation of covalent crosslink formation by a Michael-type, click-reaction between a thiol and an acrylate functionality. Different feed ratios of functional monomers resulted in a different crosslink density of the hydrogel network, allowing customization of the hydrogel’s mechanical properties to match different tissue stiffness levels.

However, non-crosslinked, homo-P(NiPAAm) hydrogel networks, formed above the T_cp_, only possess inferior mechanical properties (around 1 kPa) [22]. These are comparable to that of brain tissue (0.1–1 kPa) but are softer compared to most native tissues like organs (1–50 kPa) and connective tissue (100–2000 kPa) [23,24]. The sol-gel phase transition is beneficial for seeding cells inside the hydrogel and for cell or drug delivery applications. In order to keep the cells inside the network at low temperature, additional crosslinking interactions in the polymer network are required besides the hydrophobic interactions at temperatures above the T_cp_. Embedding cells inside networks consisting of P(NiPAAm) allows for reversibly adjusting the cell’s micro-environment by adequately controlling the temperature [25,26,27,28,29,30]. More specifically, covalent crosslinks can be introduced into the network in order to increase the hydrogel stiffness and strength. Furthermore, mild reaction conditions are required to form covalent crosslinks in the presence of cells to guarantee cell survival. ‘Click’ chemistry refers to a set of reactions taking place under mild conditions (e.g., a physiological environment) giving high yields, without any by-products and with lower cytotoxic precursors [31]. An example of such a click reaction is a Michael-type addition between thiols and acrylates, which are both easily introduced onto different types of polymer backbones (Figure 1) [32].

Lee et al. and Cheng et al. produced hydrogels by combining NiPAAm co-polymers modified with acrylates and a four-armed macromolecule containing four thiol groups [33,34]. The four-armed crosslinker required high pH to be solubilized and is highly toxic if leached from the material. The strategy from Lee et al. and Cheng et al. was inverted by Robb et al. to eliminate the usage of the toxic crosslinker by synthesizing a thermo-responsive polymer containing a thiol functionality and instead using a diacrylate PEG polymer to perform the crosslinking [35]. Wang et al. changed the previous procedure to increase the thermo-responsive behavior of the hydrogel and incorporated both groups, thiol and acrylate, onto separate thermo-responsive polymers (Figure 1) [36]. Combining both co-polymers, at 5 wt% each, resulted in a covalently crosslinked structure with higher mechanical strength than that of the individual co-polymer solutions. Wang et al. synthesized co-polymers with different feed molar ratios of 9:1 and 12:1 of thermo-responsive monomers to functionalized monomers, and confirmed incorporation of the co-monomers into the co-polymer consistent with their feed composition. From these co-polymers, they were able to produce hydrogels with a maximum storage modulus of 1.5 kPa as determined by oscillatory shear measurements at 37 °C. Further, the hydrogel’s potential as a drug delivery system was assessed by investigating its swelling and shrinking kinetics.

Bearat et al. used similar polymers to produce covalently crosslinked networks (Figure 1). However, different feed ratios were used for each individual polymer. The acrylate functionality was synthesized with a molar feed ratio of 5% and the thiol functionality with a molar feed ratio of 2% [37]. A decrease in incorporation ratio was both observed for the acrylate and thiol functionality compared to the feed ratio. Due to this mismatch in molar ratio both solutions, each at 30 wt%, were mixed at a ratio of 0.48:1 to allow stoichiometric mixing. Rheology measurements at 20 °C resulted in a storage modulus just above 1 kPa, which increased to values just below 10 kPa at 37 °C, above the transition temperature of the hydrogel. A continuous measurement wherein the temperature was gradually increased was conducted. However, it was mentioned that measurement artifacts due to slippage between the gel and the plates of the rheometer during temperature increases, caused by deswelling of the hydrogel, resulted in an underestimation of the mechanical properties of the hydrogel at high temperatures. In follow-up research, the polymers were modified with the more reactive vinyl sulfone functional group replacing the acrylates in the click reaction, thereby obtaining a higher reaction rate and slightly better mechanical properties [38]. Additionally, more extensive biocompatibility tests were performed using mouse fibroblasts for potential application in the field of endovascular embolization [39].

Based on previous work by Wang et al. [36] and Bearat et al. [37], it remains unclear which feed ratios of incorporated thiol and acrylate functionalities yield the stiffest hydrogels while conserving their thermo-responsive behavior. In our research, co-polymers with different feed ratios were synthesized yielding a library of different hydrogels, ranging from loosely to tightly crosslinked. The impact of the crosslink density on their mechanical properties was investigated in a discrete way, at 4 °C and 45 °C, to eliminate the previously reported artifacts, caused by water expulsion. From these results, the stiffest hydrogels which still remain sufficiently thermo-responsive were selected. Furthermore, uniaxial compression-relaxation tests were performed in order to better understand the time dependent behavior of the produced hydrogels. Chondrocytes were successfully embedded inside the hydrogels and their viability was assessed after 1, 3, and 7 days of incubation. Ultimately, this hydrogel has the potential to be used as a 3D cell culture with temperature modulable mechanical properties to address the scientific question of how a different micro-environment affects the behavior of the embedded cells.

## 2. Materials and Methods

### 2.1. Materials

N-isopropylacrylamide (NiPAAm), hydroxyethyl acrylate (HEA), and cysteamine hydrochloride (Cys.HCl) were purchased from TCI Europe, Zwijndrecht, Belgium. Heptane, triethylamine (N(Et)3), methanol, diethyl ether, calcium hydride, and anhydrous dichloromethane (DCM) were purchased from Acros Organics, Geel, Antwerp, Belgium. Azobisisobutyronitrile (AIBN) and 5,5′-dithio-bis-(2-nitrobenzoic Acid) (DTNB) were purchased from Merck Life Science, Darmstadt, Germany. Dithiothreitol (DTT) was purchased from Fluorchem limited, Hollingworth, UK. Acryloyl chloride, antibiotic antimycotic solution, and D_2_O were purchased from Sigma–Aldrich, Overijse, Belgium. N-Acryloxysuccinimide was purchased from BLDPharm, Shanghai, China. Anhydrous tetrahydrofuran (THF) was purchased from Honeywell specialty chemicals, Charlotte, NC, USA. Acetic acid, 1% L-glutamine solution (200 mM), and an Invitrogen™ LIVE/DEAD™ cell imaging kit were purchased from Thermofisher, Waltham, MA, USA. DMEM/F-12 was purchased from Gibco, Grand Island, NY, USA. Fetal bovine serum (FBS) was purchased from Biowest, Nuaillé, France. Visking dialysis membrane with a molecular weight cut-off of 3.5 kDa was purchased from Medicell Membranes, Londen, UK. A sterile GP Filter with a pore size of 0.22 µm was purchased from Millex, Darmstadt, Germany. Human immortalized chondrogenic cell line C28/I2 was purchased from Merck Millipore, Darmstadt, Germany, and maintained in monolayer culture containing cell culture medium at 37 °C and in a 5% CO_2_ humidified atmosphere. Composition cell culture medium: DMEM/F-12, 10 V% FBS, 1 V% L-Glutamine, and 1 V% antibiotic antimycotic solution. NiPAAm was recrystallized in heptane and AIBN was recrystallized in methanol prior to usage. Triethylamine was dried over calcium hydride and distilled.

### 2.2. Polymer Synthesis

#### 2.2.1. P(NiPAAm-co-HEAacr)

**Scheme 1:**
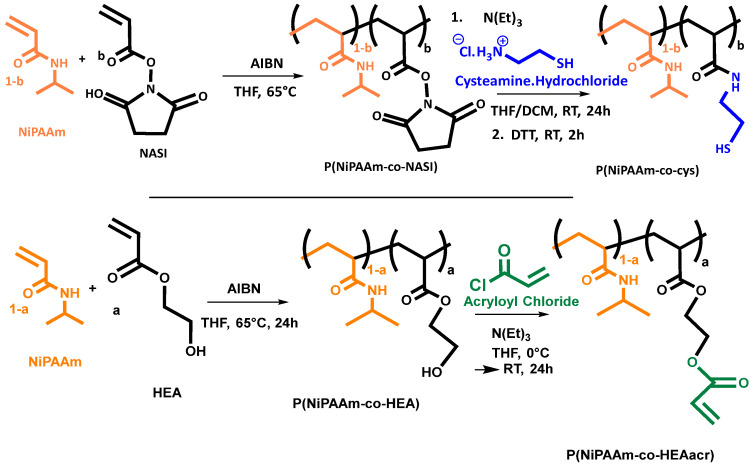
On **top**, the synthetic route to synthesize P(NiPAAm-co-Cys). **Below**: the synthetic route to synthesize P(NiPAAm-co-HEAacr).

As visualized in Scheme 1, synthesis was performed on a 25 g scale in a one-pot, two-step reaction based on a synthesis reported by Wang et al. [36] and Bearat et al. [37] (Scheme 1). In the first step, NiPAAm (25 g, 221 mmol) was dissolved at 5 wt% in anhydrous THF together with HEA in different molar ratios, ranging from 1% to 10% (0.234 mL, 2.23 mmol, for 1 mol% of HEA—2.58 mL, 24.5 mmol, for 10 mol% of HEA). The solution was degassed by bubbling with N_2_ to remove oxygen from the reaction mixture. The free radical copolymerization was initiated by adding AIBN in 0.7% equivalents to the total amount of monomers (257 mg, 1.56 mmol for 1 mol% of HEA—282 mg, 1.72 mmol for 10 mol% of HEA). The reaction mixture was heated to 65 °C for 24 h under a nitrogen atmosphere. For the second reaction step, the reaction flask was wrapped in aluminum foil and cooled down in an ice bath. Triethylamine (10 equivalents to the amount of alcohol groups on the polymer chain i.e., 3.12 mL, 22.4 mmol, for 1 mol%—34.4 mL, 246 mmol, for 10 mol%) and acryloyl chloride (1.81 mL, 22.4 mmol, for 1 mol% of HEA—19.9 mL, 246 mmol, for 10 mol% of HEA) were slowly added. The reaction mixture was stirred overnight at room temperature. Afterwards, triethylammonium chloride was removed by filtration and the filtrate was precipitated in a large excess of cold diethyl ether (2 L). The precipitated product was filtered and washed 5 times using 250 mL of cold diethyl ether. For further purification, the product was dissolved in 250 mL of an aqueous solution containing 5 V% acetic acid at 4 °C and poured in a dialysis membrane (MWCO = 3.5 kDa). The dialysis liquid, 4 L at 4 °C, was refreshed every day and the concentration of acetic acid was gradually lowered for the first three days. The following two days pure DI water was used. The entire dialysis procedure was carried out at 4 °C. After five days, the solution was frozen and lyophilized affording pure P(NiPAAm-co-HEAacr). The obtained product was weighed and an overall yield of the two-step reaction was determined (Table 1).

#### 2.2.2. P(NiPAAm-co-Cys)

As visualized in Scheme 1, a two-step reaction was performed on a 25 g scale based on a synthesis reported by Wang et al. [36] and Bearat et al. [37] (Scheme 1). In the first step NiPAAm (25 g, 221 mmol) was dissolved in 250 mL anhydrous THF together with NASI in different molar ratios, ranging from 1% to 10% (0.377 g, 2.23 mmol, for 1 mol% of NASI—4.15 g, 24.5 mmol, for 10 mol% of NASI). The solution was degassed by bubbling with N_2_ to remove oxygen from the reaction mixture. The free radical copolymerization was initiated by adding AIBN, 0.7% equivalents to the total amount of monomers (257 mg, 1.56 mmol, for 1 mol% of NASI—282 mg, 1.72 mmol, for 10 mol% of NASI). The reaction mixture was heated to 65 °C for 24 h under a nitrogen atmosphere. The reaction mixture was precipitated in a large excess, of cold diethyl ether (2 L). The precipitated product was filtered and washed 5 times using 250 mL of cold diethyl ether. The obtained product, P(NiPAAm-co-NASI) was kept under vacuum prior of using it in the post-polymerization reaction.

Cysteamine hydrochloride, 2 equivalents relative to the amount of succinimide on the polymer chain (0.508 g, 4.49 mmol, for 1 mol% of NASI—5.83 g, 51.5 mmol, for 10 mol% of NASI), was dissolved at 2 wt% in anhydrous DCM. A stoichiometric amount of triethylamine (0.625 mL, 4.49 mmol, for 1 mol% of NASI—7.18 mL, 51.5 mmol, for 10 mol% of NASI) was added. The mixture was stirred overnight resulting in a clear solution. The copolymer obtained in the previous step was separately dissolved in anhydrous THF at 5 wt%. Both solutions were combined and allowed to react overnight at room temperature under a nitrogen atmosphere. Afterwards, a stoichiometric amount of DTT to the cysteamine hydrochloride (0.692 g, 4.49 mmol, for 1 mol% of NASI—7.95 g, 51.5 mmol, for 10 mol% of NASI) was added and the reduction was allowed to occur for 2 h. Prior to precipitation in 2 L cold diethyl ether, the excess of DCM was removed by evaporation. The precipitated product was filtered and washed using 250 mL of cold diethyl ether. For further purification, the product was dissolved in 250 mL of an aqueous solution containing 5 V% acetic acid at 4 °C and poured in a dialysis membrane (MWCO = 3.5 kDa). The dialysis liquid, 4 L at 4 °C, was refreshed every day and the concentration of acetic acid was gradually lowered for the first three days. The following two days pure DI water was used. The entire dialysis procedure was carried out at 4 °C. After five days, the solution was frozen and lyophilized affording pure P(NiPAAm-co-Cys). The obtained product was weighed and an overall yield of the two-step reaction was determined, (Table 1).

### 2.3. Polymer Characterization

#### 2.3.1. ^1^H NMR Spectroscopy

60 mg of polymer was dissolved in 600 µL of D_2_O and TMS was used as a standard. The solubility of HEAacr5% was too low and the concentration was decreased from 0.1 wt% to 0.025 wt%. A ^1^H-NMR spectrum was measured on a 400 MHz spectrometer in Fourier-transform mode (Bruker Avance III, Ascend Tm 400, Billerica, MA, USA). ^1^HNMR was used to determine the molar incorporation ratio of both co-polymers by comparing the intensity of a proton peak specific to the functional monomer with a proton specific for NiPAAm. The proton on the tertiary carbon of the isopropyl group was chosen as the reference proton specific for NiPAAm at 3.91 ppm. The four protons on the ethylene backbone for both functional monomers could easily be distinguished at 4.25 ppm for P(NiPAAm-co-HEAacr) and at 2.69 and 3.39 ppm for P(NiPAAm-co-Cys). The protons at 6.05, 6.24 and 6.46 ppm of the acrylate group of P(NiPAAm-co-HEAacr) were used as well, as an indication of the conversion of the post-polymerization reaction. Calculations and spectra can be found in the Supporting Information.

#### 2.3.2. FTIR

FTIR measurements were performed on a Bruker alpha IR spectrometer (scans: 32, resolution: 4 cm^−1^, wavenumber range: 4000–400 cm^−1^).

#### 2.3.3. Size-Exclusion Chromatography (SEC)

SEC was performed on an Agilent 1260-series HPLC system, Santa Clara, CA, USA, equipped with a 1260 online degasser, a 1260 ISO-pump, a 1260 automatic liquid sampler (ALS), a thermostated column compartment (TCC) at 50° C equipped with two PLgel 5 mm mixed-D columns and a guard column in series. SEC is coupled with the following detectors: a 1260 diode array detector (DAD), a 1260 refractive index detector (RID), and a multi-angle light scattering detector (Wyatt miniDawn Treos II, Santa Barbara, CA, USA). The refractive index (RI) increment (dn/dc) values were determined via the in-line RI detector. The MALS-RID data was further analyzed with the Astra 7.0.1.24 software, from Wyatt Technology, Santa Barbara, CA, USA. The used eluent was N,N-dimethylacetamide DMA containing 50 mmol of LiCl at a flow rate of 0.500 mL/min. The spectra were analyzed using the Agilent Chemstation software, version 3.6.2.4., with the GPC add-on.

#### 2.3.4. Ellman’s Method

3 mg of P(NiPAAm-co-Cys) was dissolved at 0.10 wt% in a PBS-buffer (pH = 7.4, 0.1 M). 1.8 mL of a 5 mM 5,5-dithio-bis-(2-nitrobenzoic acid) (DTNB) solution in PBS-buffer (pH = 7.4, 0.1 M) was added. Free thiols on the polymer chain of P(NiPAAm-co-Cys) will react with DTNB, forming 2-nitro-5-thiobenzoic acid (TNB) which ionizes in PBS to the TNB^2−^ ion. The absorbance of TNB^2−^ was measured with UV-VIS (Agilent, Varian Cary 6000, Santa Clara, CA, USA). The concentration of free thiols can be calculated via the Lambert-Beer law with extinction coefficient of 14,150 M^−1^ cm^−1^ at a characteristic wavelength of 412 nm with the DTNB solution as a reference.

#### 2.3.5. Solubility

Polymers were solubilized over 8 h in cell culture medium at 4 °C at different weight percentages. The maximum solubility was determined as the solution with the highest possible weight percentage which can still be passed through a sterile filter with pore size 0.22 µm.

#### 2.3.6. Differential Scanning Calorimetry (DSC)

Measurements were performed on 10 µL samples of 5 wt% polymer solutions in cell culture medium dropped into a tared aluminum cup, which was sealed with an aluminum hermetic lit to prevent water from evaporating. Differential scanning calorimetry measurements were performed using a TA instruments, DSC Q2000, New Castle, DE, USA. Four cycles were performed on the same sample. One cycle consists of heating from 2 °C to 50 °C at a rate of 5 °C/min and cooling back down to 2 °C at the same rate of 5 °C/min. Between each cycle an isothermal period of 30 min was maintained to allow the solution to fully equilibrate. Three samples were measured for each different co-polymer. The cloud point (T_cp_) of the solution was determined as the onset of an increase in the heat flow. The temperature at the maximum heat flow is reported as T_max_.

### 2.4. Hydrogel Formulation

Synthesized co-polymers with different ratios of incorporated functional co-monomer were dissolved at the maximum solubility of the least soluble co-polymer, HEAacr, in cell culture medium. The mixtures were gently centrifuged, to limit the formation of air bubbles and cooled down to enhance dissolution. Once dissolved, the polymer solutions were sterilized by passing them through a sterile filter with pore size of 0.22 µm. At this stage, only for cell viability studies, the sterile Cys-polymer solutions was used to resuspend a pellet of a known amount of chondrocytes and gently mixed to guarantee homogenous cell embedding. Afterwards, the solutions were loaded into a syringe, air bubbles were carefully removed by putting the syringe upright and pushing out the formed air bubbles through the needle and the solutions were cooled down to 4 °C. Both syringes were coupled through a small plastic piece and the solutions were pushed back and forth between the syringes for 20 times. Three different administration Methods were developed. Method 1: Immediately after mixing, injection of the polymer mixture was confirmed using a 23 G needle, illustrated by a movie in Supporting Information, Movie S1. Method 2: A small droplet was pushed out of the syringe and spread to obtain a flattened droplet. Method 3: To form discs, the polymer mixture was first left to react inside the syringe at room temperature for 30 min, followed by 30 min at 37 °C. Discs were cut from the syringe using a box cutter at room temperature, having a height of approximately 2 to 3 mm and a diameter of 1 cm depending on the volume of the used syringe.

### 2.5. Hydrogel Characterization

#### 2.5.1. Gel Fraction (GF)

Four small hydrogel discs, as prepared by method 3 in Section 2.4, were dried in a desiccator over silica gel beads at room temperature immediately after being formed in the syringe. After five days, the masses of the dried hydrogel discs were determined. Afterwards, the hydrogel discs were incubated in 10 mL cell culture medium at 37 °C. The incubation medium was refreshed twice a day for 3 days. The washed hydrogel discs were again placed in a desiccator for five days. The dried hydrogel discs were weighed. The gel fraction was calculated by dividing the mass of the dried hydrogel after and before the washing step.

(1) $$GF=\frac{m(dried\;hydrogel\;after\;washing)}{m(dried\;hydrogel\;before\;washing)}$$

#### 2.5.2. Swelling Degree (SD)

Hydrogel discs, as prepared by method 3 in Section 2.4, were incubated at different temperatures for at least one hour to guarantee that the swelling reached an equilibrium. Water droplets on the surface of the hydrogel discs were removed before weighing the disc. This process was repeated at different pre-defined temperatures. Afterwards, hydrogels were incubated for five days in a desiccator over silica gel beads at room temperature and the weight of the dried hydrogel was determined. The swelling degree can be calculated by following equation:

(2) $$SD=\frac{m\left(swollen\;hydrogel\;at\;temp.T\right)-m(dried\;hydrogel)}{m(dried\;hydrogel)}$$

### 2.6. Rheology

Hydrogels, as prepared by method 3 in Section 2.4, were punched into discs with a diameter of 8 mm and placed between the parallel plates of an Anton Paar MCR702 rheometer, Gent, Belgium. The plates were sandblasted to avoid slippage between the plate and the sample. The lower plate of the rheometer was set at a specific temperature. Prior to the measurement, hydrogels were first incubated for at least 1 h at the desired temperature. After loading, a normal force of 0.05 N (for 4 °C) and 0.25 N (for 45 °C) was applied on the hydrogels to guarantee full contact between the plates and the hydrogels. For intermediate temperatures, the applied normal force ranged between 0.05 N to 0.25 N. Discs were surrounded by mineral oil to prevent water from evaporating out of the hydrogel during measurements. Oscillatory shear was applied at a frequency of 1 Hz and a strain of 1% to determine the equilibrium storage (G′) and loss (G″) moduli. A frequency of 1 Hz was selected to mimic a human’s step frequency and a strain of 1% was determined to be in the linear visco-elastic region of the material. Further, a frequency sweep was executed in the frequency range of 0.0159 Hz to 100 Hz at a strain of 1%. A strain sweep was executed wherein the strain is gradually increased from 0.01% to 1000% at 1 Hz. Degradation over time was assessed by determination of G*, which is the modulus of G′ + G″, of the hydrogels at 4 °C after being incubated at 37 °C for 3 and 7 weeks.

### 2.7. Compression-Relaxation

Hydrogels, as prepared by method 3 in Section 2.4, were incubated overnight at either 4 °C or 45 °C in cell culture medium. The diameter and height of the hydrogels were determined prior to the measurement (in house developed Micro Laser Scanner in collaboration with Acacia Technology Schilde, Antwerp, KU Leuven Core Facility for Biomechanical Experimentation). Hydrogel discs were carefully placed between two parallel plates (Bose ElectroForce, TA Instrument company, New Castle, DE, USA.) while being incubated in cell culture medium at either 4 °C or 45 °C. An unconfined compression-relaxation test was performed, where a maximum strain of 20% ($\varepsilon_{max}$) of the initial sample height was applied to the hydrogel discs at a rate of 10%/sec followed by a 900 s relaxation. The reaction force was recorded during the compression-relaxation experiment. The maximal force ($F_{max}$) is defined as the highest measured force, after two seconds. The relaxed force ($F_{relax}$) is the force at the end of the measurement when sufficient time for relaxation was allowed. The respective engineering stresses ($\sigma_{comp.}$ and $\sigma_{relax}$) and moduli ($E_{comp.}$ and $E_{relax}$) are defined according to following equations:

(3) $$\sigma_{comp.}=\frac{F_{max}}{A{rea}_{undeformed}};\sigma_{relax}=\frac{F_{relax}}{A{rea}_{undeformed}}$$

(4) $$E_{comp.}=\frac{\sigma_{comp.}}{\varepsilon_{max}};E_{relax}=\frac{\sigma_{relax}}{\varepsilon_{max}}$$

### 2.8. Cell Viability

C28/I2 chondrocytes were used to assess cell viability inside the flattened hydrogel droplet at a concentration of 0.5 million cells/mL. The concentration of the used polymer in cell culture medium was decreased to 5 wt% for both Hydr2% and Hydr3% to limit the auto-fluorescent background produced by the hydrogel during imaging, which was interfering at the wavelength of the used dyes. The cell-containing hydrogels, as prepared by method 2 in Section 2.4, were imaged after 1, 3 and 7 days of incubation. Prior to imaging, the hydrogels were washed twice with PBS. Afterwards, the hydrogels were stained using Invitrogen™ LIVE/DEAD™ cell imaging kit according to the manufacturer’s directions in FBS-free cell culture medium and incubated for 30 min at 37 °C. The hydrogels were washed three times with PBS and were imaged with a Leica SP8 inverted confocal microscope with an HC PL APO 10×, 0.4 numerical aperture dry objective. The living cells were visualized in green using Calcein AM (488/515) and the dead cells were visualized red using BOBO-3 Iodide (570/602). The cell viability was determined by dividing the amount of living cells by the total amount of cells. (frames: 1200 µm × 1200 µm, *n* = 5).

## 3. Results and Discussion

### 3.1. Polymer Synthesis and Characterization

The two-step synthesis to obtain both P(NiPAAm)-based reactive copolymers, P(NiPAAm-co-HEAacr) and P(NiPAAm-co-Cys), have already been described by Wang et al. [36] and Bearat et al. [37]. Both groups were able to successfully produce hydrogel networks by combining these two polymer solutions. Nevertheless, optimization of the ratio of functional monomer to thermo-responsive monomer yielding more robust hydrogels was never executed.

In this paper, co-polymers were synthesized with different molar ratios of functional monomer to thermo-responsive monomer. Initially, the molar feed percentages ranges from 1% to 10%. However, due to the decreasing solubility of the obtained co-polymers, only the results obtained for co-polymers with functional monomer ratios ranging from 1% to 5% will be discussed further.

#### 3.1.1. Incorporation Ratio

^1^HNMR spectroscopy was used to calculate the incorporation ratio of both co-monomers by comparing the intensity of a proton peak specific to the functional monomer with a proton specific for NiPAAm. Spectra and calculations are shown in Figures S1 and S2 and Tables S1–S4 in the Supporting Information. An overview of the results is given in Table 1. The percentage following the name of the co-polymer indicates the feed ratio of functional monomer. From these results, an increasing incorporation of functional monomer with increasing feed ratio is confirmed and both percentages match closely. The solubility of co-polymers containing HEAacr decreases with HEAacr concentration, preventing to obtain an accurate ^1^HNMR spectra for HEAacr5. The Ellman’s method was used to calculate the amount of free thiols present on P(NiPAAm-co-Cys) [40]. (Table 1 and Table S5 in the Supporting Information), are comparable to the results obtained with ^1^HNMR spectroscopy, indicating that the reduction step is performed with high yields. The molecular weight and dispersity (Table 1) were measured using SEC. For Cys1 and HEAacr1 a normal distribution was observed. Comparable molecular weights of 11.4 × 10^3^ g/mol and 11.5 × 10^3^ g/mol and a rather low dispersity of 1.4 were measured. Increasing the amount of functional monomers increased the dispersity for both Cys and HEAacr. This effect was only minor for the Cys co-polymer. However, in the case of the HEAacr polymers a second peak with a higher molar mass was observed in the LS spectra before the main peak, which increased the overall dispersity and molar mass. It is postulated that due to a decrease in solubility, aggregation of the polymer in DMA (Eluent) is induced resulting in higher apparent molar masses, although it cannot be excluded that a very small amount of crosslinked fraction was formed. For small ratios of functional monomer, HEAacr2% and HEAacr3%, this higher molar mass peak corresponds to approximately 1 wt% or less of the injected sample. For higher incorporation ratios of 4% and 5% of HEAacr the results are not reliable enough to draw any conclusions due to their low solubility. The successful preparation of the target polymers was also confirmed by FTIR spectroscopy (Supporting Information, Figures S3 and S4).

#### 3.1.2. Solubility of Polymers

**Figure 2:**
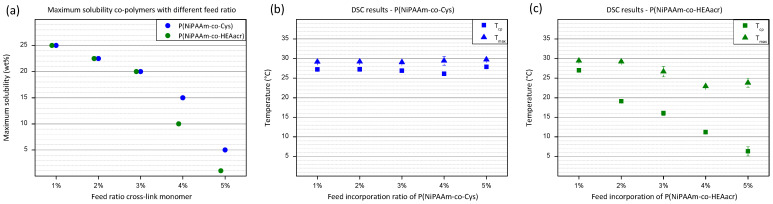
(**a**) The maximum solubility of polymer solutions was plotted in function of feed ratio of functional monomer. (**b**) Results obtained from DSC measurement for P(NiPAAm-co-Cys) with different feed ratio. (**c**) Results obtained from DSC measurement for P(NiPAAm-co-HEAacr) with different feed ratio. T_cp_ = cloud point temperature, the onset of an increase in the heat flow. T_max_ = maximum temperature, the temperature at the maximum heat flow. Error bars: standard deviation.

At 1% incorporation ratio of functional monomer, both co-polymers are very-well soluble, with a maximum solubility of 25 wt% in cell culture medium. Solubility tests were performed in cell culture medium, in view of embedding cells inside the hydrogel. An overall decrease in solubility is observed for both co-polymers with increasing amount of functional monomer incorporated into the polymer chain (Figure 2a). The higher hydrophobic nature of P(NiPAAm-co-HEAacr) compared to P(NiPAAm-co-Cys), has a bigger influence on the solubility when higher incorporation ratios are reached. A sudden decrease in solubility is observed for the P(NiPAAm-co-HEAacr) (green) above an incorporation ratio of 4%, reaching a solubility of only 5 wt%. At a ratio of 5%, the solubility further decreases to 1 wt%. The decrease is less dominant for P(NiPAAm-co-Cys) (blue), with the lowest solubility of 5 wt% at an incorporation ratio of 5%. Co-polymers with higher incorporation ratios than 5% will not be discussed due to their low solubility. Hydrogels will be produced with polymer solutions based on the maximum solubility of the least soluble polymer, P(NiPAAm-co-HEAacr).

#### 3.1.3. Thermo-Responsive Behavior of Polymer Solution

At low temperatures, the polymers are soluble in the aqueous phase caused by favorable interactions with water molecules. Increasing the temperature will decrease their affinity with water, until a transition temperature is reached, called the cloud point temperature (T_cp_), where polymers start to phase separate due to dehydration. The heat needed for this transition is measured using DSC [20].

The T_cp_ of P(NiPAAm-co-Cys), remains around 26 °C for all incorporation ratios (Figure 2b). The same is observed for the temperature at the maximum heat flow (T_max_), remaining around 29 °C. In contrast, T_cp_ decreases severely for P(NiPAAm-co-HEAacr) with increasing incorporation ratio (Figure 2c). At 1%, a T_cp_ of 26 °C is measured, similar to P(NiPAAm-co-Cys). Increasing the ratio to 5%, gradually decreases the T_cp_ to 6 °C. The decrease for the T_max_ is less outspoken, with T_max_ decreasing from 29 °C to 24 °C. This indicates that the transition region broadens with increasing functional monomer percentage for P(NiPAAm-co-HEAacr). The functional monomer HEAacr has a much bigger influence on the solubility and on the cloud point temperature compared to the Cys functional monomer due to the hydrophobic nature of HEAacr [41,42].

### 3.2. Hydrogel Formulation and Characterization

Both co-polymers consist of the same thermo-responsive monomer, NiPAAm, and a different functional monomer, either HEAacr or Cys. By mixing both polymer cell culture medium solutions, in view of embedding cells inside the hydrogel, a spontaneous crosslink reaction occurs in the form of a Michael-type addition between the acrylate group of HEAacr and the thiol group of Cys. Hydrogels were formed by combining co-polymer solutions with the same incorporation ratio of functional monomers and both co-polymers were dissolved at the maximum solubility of the least soluble co-polymer to allow stoichiometric mixing. The produced hydrogels are named Hydrx%, the percentage ‘x’ following Hydr indicates the feed ratio of functional monomer used. Hydrogels were produced from co-polymers with different feed ratio ranging from 1% to 5%. Due to the low solubility of HEAacr5, very unstable gels were obtained, which will not be discussed further. A higher concentration of functional monomer on the polymer chain will result in a more densely crosslinked network. Oppositely, the thermo-responsive behavior of the hydrogel will be suppressed by this tightly crosslinked network. Increasing the incorporation ratio of functional co-monomers will increase the amount of crosslink functionalities in solution. However, as the maximum solubility of the polymer chains decreases with increasing incorporation ratio a lower overall amount of crosslink functionalities can be dissolved above 3% of functional monomers. An optimal ratio of functional monomers can be determined, as a sufficiently high incorporation ratio to produce a densely crosslinked network which is not too high to decrease the solubility of the co-polymer, yielding the most robust hydrogels possible.

Different administration methods can be performed, making this gelation system compatible with different applications and characterization methods. Discs were used for mechanical characterization of the hydrogel and will be used in further research regarding the changes in the cell’s behavior to altered mechanical micro-environments [43,44,45,46]. Chondrocytes were chosen in this study as they are known to change their phenotype upon changes in their micro-environment [47,48,49,50,51]. The best resolution for microscopy was obtained by imaging flattened droplets embedded with chondrocytes, allowing to assess the cell viability of the hydrogel. Lastly, the injectability of the system was confirmed through a 23 G needle opening a wide array of applications. However, it should be noted that injection should be performed in approximately less than 60 s after the start of mixing due to the fast crosslinking reaction (Movie S1).

#### 3.2.1. Gel Fraction

**Figure 3:**
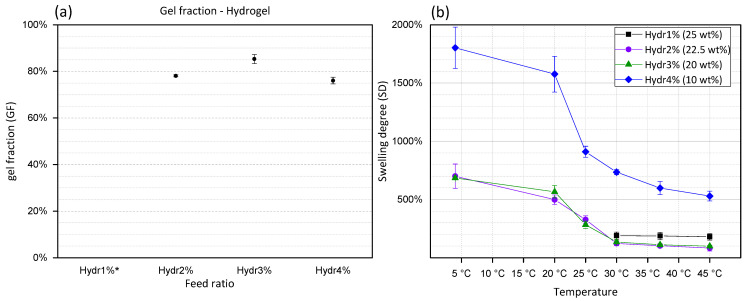
(**a**) Gel fraction of hydrogels in function of feed percentage of functional monomer. * Hydr1% is too unstable to obtain accurate results. (**b**) Swelling degree (SD) of hydrogels consisting of polymers with different feed ratio. Hydr1% (25 wt%) in black, Hydr2% (22.5 wt%) in purple, Hydr3% (20 wt%) in green and Hydr4% (10 wt%) in blue. Error bars: standard deviation.

The gel fraction (GF) is calculated by dividing the mass of the dried polymer network before and after washing of the hydrogel. During this washing step, unreacted polymer chains will diffuse out of the hydrogel network. GF is a representation of the amount of formed covalent crosslinks between different polymers. From Figure 3a, it is concluded that Hydr2% (78%) and Hydr4% (76%) have a high gel fraction which confirms the high efficiency of the Michael-type crosslinking reaction. The highest gel fraction (85%) was obtained for Hydr3%, indicating that at a 3% incorporation ratio the most efficient crosslinking reaction occurred. Hydr1%, was insufficiently stable at low temperatures to perform this type of measurements due to the small amount of covalent crosslinks.

#### 3.2.2. Swelling Degree

Represents the amount of water inside the hydrogel network. By measuring the swelling degree at different temperatures, the thermo-responsive behavior can be visualized. At low temperatures the highest SD are measured. By increasing the temperature, NiPAAm co-monomers become less soluble and will interact with fewer water molecules which results in a decrease of the swelling degree [52].

Similar behavior is observed for all hydrogels with different feed ratios of functional monomers (Figure 3b). A high swelling degree is observed at 4 °C. Increasing the temperature, gradually decreases the SD with the largest change in SD between 20 °C and 30 °C. Above 30 °C, a plateau is observed and the hydrogels turned opaque. In contrast to the T_cp_, Section 3.1.3, no significant influence on the transition temperature was observed by comparing hydrogels produced from polymers with different percentage of functional co-monomers. The nature of the polymer network is different compared to a polymer in solution. The T_cp_ measured using DSC indicates a coil-to-globule transition [53]. Whereas for the polymer network the coil phase does not exist and only a change of affinity towards water molecules can be observed by measuring the SD in function of temperature [52]. Hydr1% shows a slightly higher SD at high temperatures due to the low number of crosslinks formed. The gels become very unstable at temperatures lower than 30 °C, making it impossible to obtain accurate results. The SD of Hydr4% in function of temperature shows the same trend as that of Hydr2% and Hydr3%. However, a much higher overall SD is measured, caused by the preparation of the hydrogels at lower polymer concentration due to the lower solubility of HEAacr4.

### 3.3. Mechanical Properties

By mixing both polymers, covalent bonds are spontaneously formed by a Michael-type ‘click’ reaction. At low temperature, these covalent crosslinks build up the polymer network. By increasing the temperature, additional hydrophobic interactions are formed in the covalent crosslinked polymer network and will drastically change the nature of the hydrogel network.

To investigate these changes, other research groups already performed measurements wherein the temperature was continuously increased while monitoring changes in mechanical properties [37,38]. However, artifacts were present in these measurements, caused by the deswelling of the hydrogel when being heated. Water was expelled out of the hydrogel and ended up between the hydrogel and the apparatus thereby initiating slippage. This slippage resulted in an underestimation of the mechanical properties of the hydrogel at higher temperatures. Another aspect is the constant force applied on the hydrogel which restricted the hydrogel from reaching its equilibrium swelling degree.

In our research, the mechanical properties of the hydrogels were investigated both at 4 °C and at 45 °C after incubation of the hydrogel samples at the corresponding temperature. These temperatures were chosen to be well above and below the cloud point temperature of P(NiPAAm). By performing these discontinuous measurements in function of temperature, artefacts of heating the samples are eliminated. Two different types of loading were applied, to obtain a better understanding of the time dependent behavior of the hydrogel material. First, the response to an oscillatory shear stress applied in rheology experiments is discussed. Secondly, the results obtained from uniaxial compression followed by relaxation is discussed.

#### 3.3.1. Rheology: Different Incorporation Ratios

**Figure 4:**
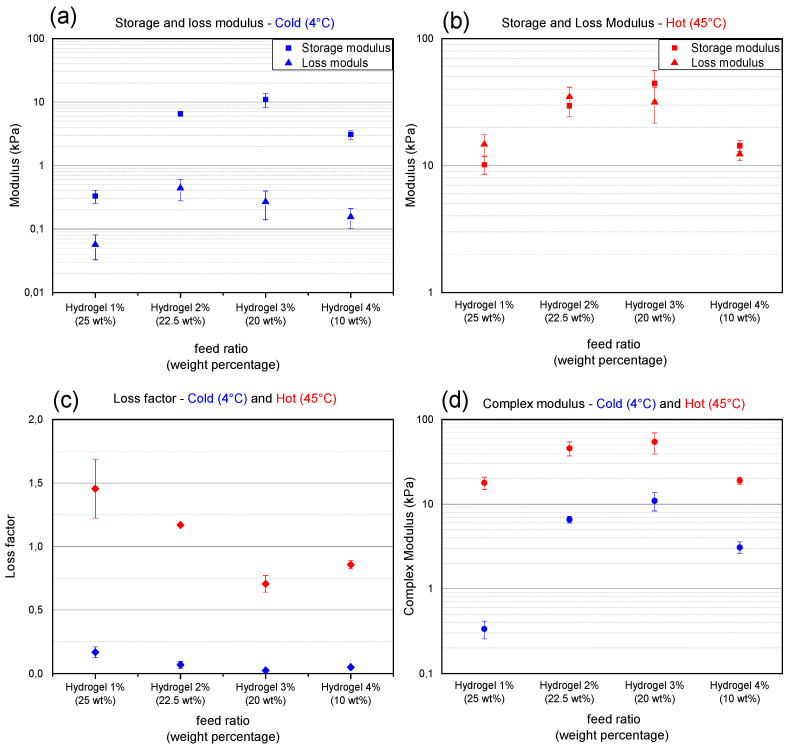
*X*-axis represents hydrogels consisting of polymers with a different incorporation ratio and dissolved at maximum solubility between brackets. Points depicted in blue are measured at 4 °C, red points at 45 °C. (**a**,**b**) Storage modulus (squares) and loss modulus (triangles). (**c**) Loss factor. (**d**) Complex modulus. Error bars: standard deviation. All measurements were performed at a frequency of 1Hz and 1% strain. This data is summarized in Table S6.

The mechanical properties of hydrogels consisting of co-polymers with different incorporation ratios of functional monomers are compared both at 4 °C and 45 °C. At 4 °C, a clear increase in storage modulus, which represents the elastic properties, is initially observed by increasing the incorporation ratio of functional monomer (Figure 4a). By increasing the amount of available crosslinks, hydrogel networks with a higher cross-link density are formed resulting in a higher storage modulus. The storage modulus reaches a maximum for Hydr3%. The solubility of polymers with an incorporation ratio of 4% is half of the solubility of polymers with an incorporation ratio of 3%. The low solubility of the polymer decreases the amount of available crosslink functionalities in solution thereby producing a more loosely crosslinked network with a lower storage modulus. The loss moduli, which represent the viscous properties, are low for all different hydrogels and the material can be described as elastic. From these results, it can be concluded that Hydr3% has the most densely covalently crosslinked hydrogel network with a storage modulus of 11 ± 3 kPa and a loss modulus of 0.27 ± 0.13 kPa at 4 °C. A significant increase in both storage and loss modulus can be observed when measuring the hydrogel discs at 45 °C (Figure 4b). The increase is caused by the additionally formed hydrophobic interactions which are reversible, and thus can be broken and reformed again when given sufficient time [54,55]. This changes the nature of the irreversible network present at 4 °C to a more reversible network at 45 °C. These reversible interactions make the material more viscous, which translates in a large increase of the loss modulus compared to the storage modulus. For Hydr1% and Hydr2% the loss modulus is dominant over the storage modulus indicative of viscoelastic materials in contrast to Hydr3% and Hydr4% where the storage modulus is dominant over the loss modulus [56]. At lower incorporation ratios, a higher amount of thermo-responsive monomers is present and a more outspoken thermo-responsive behavior is observed. A similar trend for the storage modulus is observed as for the cold samples, reaching a maximum at Hydr3% with a storage modulus of 44 ± 12 kPa and a loss modulus of 32 ± 10 kPa.

The change in loss factor, which is the ratio between the loss and storage modulus, clearly shows the change of the nature of the material by heating the samples above their transition temperature (Figure 4c). All samples have a low loss factor at 4 °C and can be described as elastic. The more loosely crosslinked hydrogels, Hydr1% and Hydr2%, have a loss factor bigger than one and can be described as viscous materials above their transition temperature. The more densely crosslinked hydrogels, Hydr3% and Hydr4%, have a loss factor lower than one and are described as visco-elastic above their transition temperature [56]. By comparing the complex modulus for cold and hot samples the contribution of the hydrophobic interactions at higher temperatures can be visualized (Figure 4d). The biggest difference between the complex modulus from cold and hot samples is observed for Hydr1%. The complex modulus of hot samples is 60 times bigger than that for the cold samples. This can be explained by the polymer having the highest ratio of thermo-responsive monomers and a loose network structure, permitting the most efficient dehydration and formation of physical interactions. The difference in complex modulus decreases with increasing incorporation ratio of functional monomers.

A 7-time increase is observed for Hydr2% and a 5-time increase for Hydr3%. The more loosely crosslinked hydrogel network of Hydr4% allows more physical interactions to take place, making the difference between cold and hot samples a factor 6. The most outspoken thermo-responsiveness was observed for Hydr1%, with a 60 times increase in complex modulus. However, the low mechanical properties at low temperature are insufficient to allow manipulation of the hydrogel discs. A benchmark of a storage modulus of 1kPa was set to guarantee that the discs are strong enough to withstand manipulation. The low solubility of the co-polymers used to produce Hydr4% caused the mechanical properties to decrease. Based on these results, it was decided to perform further investigation for the mechanical properties of only Hydr2% and Hydr3%.

**Figure 5:**
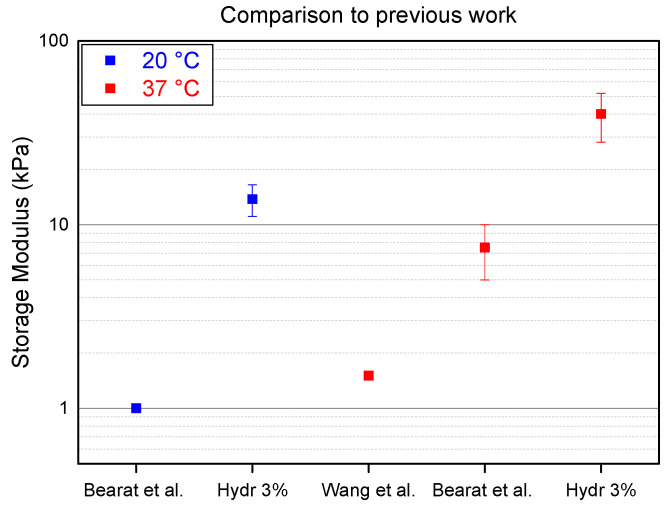
Comparison of hydrogel described in this manuscript (Hydr3%) to the state of the art (Bearat et al. and Wang et al. [36,37]) at 20 °C in blue and at 37 °C in red.

Two different research groups, Wang et al. and Bearat et al., independently synthesized polymers containing the same monomer units. However, neither of these research groups did a thorough research on the effect of the ratio of these monomer units. To illustrate how this manuscript goes beyond the state of the art, the storage modulus of the stiffest hydrogel described in this publication, Hydr3%, is compared to the stiffest hydrogels from previous work (Figure 5).

Bearat et al. synthesized a polymer containing 5% of acrylate functionalized monomer units and a polymer containing 2% of thiol functionalized monomer units [37]. Both polymers were dissolved at 30 wt% in a PBS buffer and mixed at a ratio of 0.48:1 to allow stoichiometric reaction of the acrylates and the thiols. At a temperature below the transition temperature (20 °C) Hydr3% is 10 times stiffer compared to the hydrogels produced by Bearat et al. This indicates that more covalent cross-links are formed by mixing polymers with the same monomer unit ratio which allows a more efficient stoichiometric mixing at a ratio of 1:1. At physiological temperature, Hydr3% is six times stronger than the hydrogel produced by Bearat et al.

Wang et al. synthesized polymers containing 10% of cross-link monomer units which resulted in a low solubility of only 5 wt% in a PBS buffer [36]. This low solubility only allowed to produce hydrogels with minor mechanical properties, which are around 30 times lower than those of the hydrogel described in this manuscript. These results show that the optimization of the ratio of cross-link monomers results in much stiffer hydrogels.

#### 3.3.2. Rheology: Frequency and Strain Sweep

The frequency and strain sweep will only be discussed for Hydr3% to illustrate the different nature of the hydrogel network at temperatures below the transition temperature (4 °C) and above the transition temperature (45 °C). The nature of the frequency and strain sweep of Hydr2% are almost identical to those of Hydr3% and the same conclusions could be drawn from these graphs (Figure S5).

**Figure 6:**
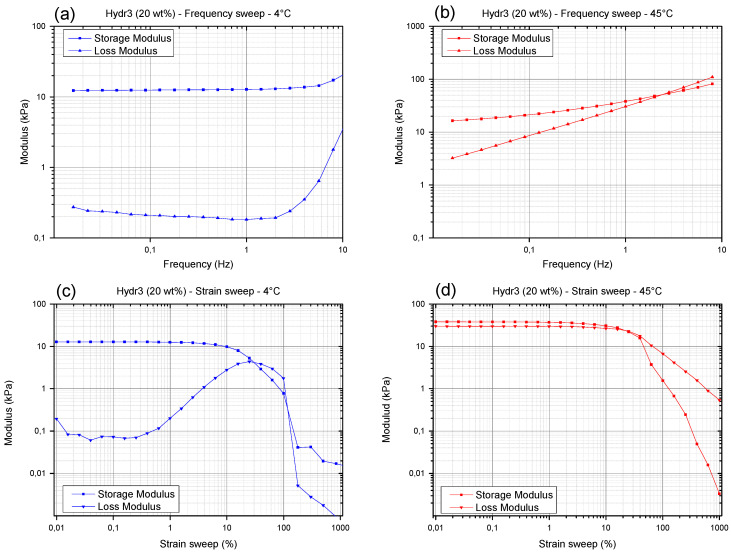
(**a**) Frequency sweep of Hydr3% at 4 °C. (**b**) Frequency sweep of Hydr3% at 45 °C. (**c**) Strain sweep of Hydr3% at 4 °C. (**d**) Strain sweep of Hydr3% at 45 °C.

At low temperature, no frequency dependent behavior can be observed, indicating that there is no relaxation of the covalently crosslinked network (Figure 6a). Only at high frequencies, most probably located within the Rouse regime probing local mobility of the chain segments, a small increase in loss and storage modulus is observed with increasing frequency. At higher temperature (Figure 6b) strong frequency dependent behavior is observed over a wide frequency range. Relaxations of the additionally formed hydrophobic interactions on larger time scales cause the loss modulus and, to a lesser extent, the storage modulus to decrease at lower frequencies [54,55]. However, there still is a significant difference between the moduli measured at high temperature and at a low frequency of 0.0159 Hz compared to a low temperature and a regular frequency of 1Hz. This means that there was not sufficient time at 0.0159 Hz, for all the reversible bonds to completely relax.

When varying the applied strain amplitude at 4 °C, a maximum in the loss modulus is observed between a strain of 10% and 100% (Figure 6c). It is postulated that initially strain stiffening occurs by stretching the semi-flexible segments between covalent crosslinks [57]. At higher deformations, polymer chains will align with the direction of deformation causing a decrease in the storage and loss modulus and eventually the sample will start to slip. At higher temperatures, strain softening was observed from strains of 10% and onwards (Figure 6d). The polymer chains aligned with the direction of flow cause the storage and loss modulus to decrease. It is postulated that by further increasing the amplitude of the shear strain first the weaker physical interactions were broken before the stronger covalent bonds experienced significant stretch. Hence a maximum in the loss modulus was not observed anymore [58].

#### 3.3.3. Rheology: Transition Temperature

**Figure 7:**
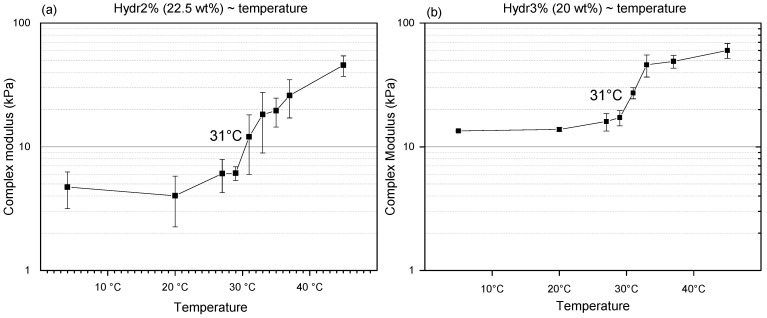
Complex modulus of (**a**) Hydr2% and (**b**) Hydr3% in function of temperature. All measurements were performed at a frequency of 1Hz and a 1% strain.

To determine the transition temperature, discontinuous measurements at different temperatures were performed on Hydr2% and Hydr3%. At low temperature, a plateau is observed for both hydrogels (Figure 7). Mechanical properties start to increase at a temperature of 29 °C and a clear transition around 31 °C can be observed. The transition temperature is different from the T_cp_ measured using DSC, because the nature of the polymers in solution is different from the crosslinked polymer network and other characteristics are being measured. For DSC, the T_cp_ is identified with a collapse of the polymer chain, whereas for the rheological properties the increase is correlated with the formation of additional hydrophobic interactions. For both measurements there also appears to be no correlation with the swelling degree in function of temperature, because again another type of interactions is being measured, namely the affinity of NiPAAm with water by determining the energetic effects for polymer dehydration with increasing temperature [52]. The transition temperature of the mechanical properties appears to be above the transition range that shows changes in swelling. It can be hypothesized that the NiPAAm monomers gradually lose all their affinity with water molecules and subsequently start to interact with each other. This also explains the more gradual change of swelling degree compared to the sharp transition observed from the mechanical properties in function of temperature. It is notable that the transition temperature is very close to the frequently reported LCST of homo-P(NiPAAm), 32 °C, which indicates that the influence of the crosslinking on this transition temperature is negligible [26]. Above the transition temperature, a plateau is again observed. For Hydr3%, the transition is much more pronounced compared to Hydr2% (Figure 7). Hydr2% is more loosely crosslinked than Hydr3%, allowing more physical interactions to be formed.

#### 3.3.4. Rheology: Degradation over Time

**Figure 8:**
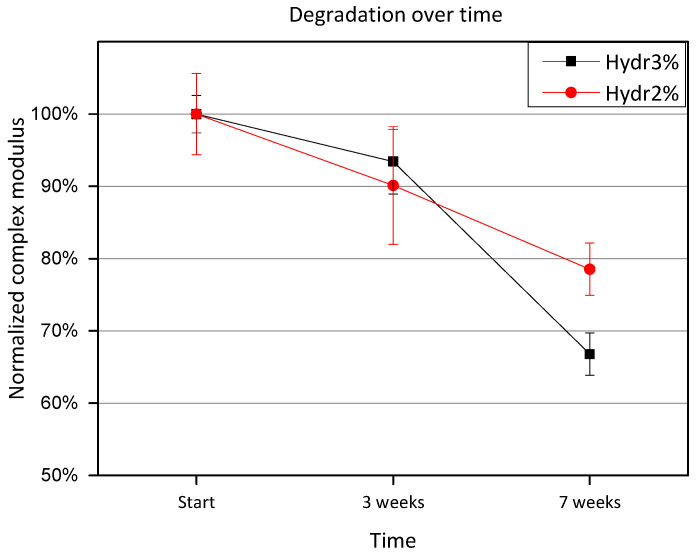
Normalized complex modulus as a function of time shows the degradation over time. The complex modulus was normalized by the complex modulus measured at the start.

Hydrogels discs were incubated at 37 °C between measurements. Measurements were performed at 4 °C, to give an indication of the amount of degraded covalent crosslinks. After 3 weeks of incubation, a small amount of degradation is observed, however no significant difference is measured for either Hydr3% or Hydr2% (Figure 8). After 7 weeks, a significant difference is observed and only 80% of the initial complex modulus is preserved for Hydr2% and 70% for Hydr3%. It is postulated that hydrolysis of the ester bonds of the crosslinker caused the degradation over time. 

#### 3.3.5. Mechanical Properties: Compression-Relaxation

**Figure 9:**
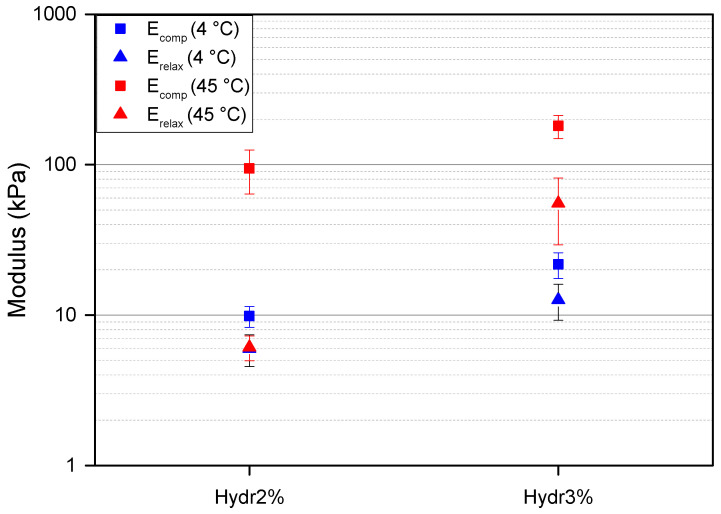
Results for the compression-relaxation experiments. Compression moduli are depicted as squares, relaxation moduli as triangles. Blue = 4 °C and red = 45 °C. The following data was summarized in Table S7.

To better understand the relaxation behavior of the hydrogel at different temperatures fast compression tests were performed followed by a long relaxation. At 4 °C, more than double the compressive force was required to achieve the same compression for Hydr3% compared to Hydr2% with a similar cross-sectional area. A compression modulus of 27 ± 5 kPa for Hydr3% and of 12 ± 2 kPa for Hydr2% were calculated (Figure 9). These results are in agreement with the obtained results from the rheology measurements, where almost a two times higher complex modulus was obtained for Hydr3% than for Hydr2% due to the more densely crosslinked network of Hydr3%. At low temperature, a small amount of relaxation, around 40%, is observed and after 900 s a plateau is observed for both hydrogels (Figure S6). This relaxation behavior is attributed to the fluid pressure which relaxes over time [59].

At 45 °C, a ten times higher compression modulus was obtained compared to the measurements at a lower temperature of 4 °C for both hydrogels because of the formation of hydrophobic interactions above the T_cp_ (Figure 9). A higher compression modulus of 230 ± 40 kPa for Hydr3% compared to 120 ± 40 kPa for Hydr2% was calculated, due to the more densely crosslinked network of Hydr3%. More than twice the amount of relaxation was observed at high temperature compared to the measurements at 4 °C due to the reversible nature of the additionally formed interactions which relax over time besides the relaxation of the fluid pressure [54,55]. The relaxation is bigger for Hydr2% (92%) compared to Hydr3% (69%) due to the higher percentage of incorporated thermo-responsive monomer in Hydr2%. A relaxation modulus was calculated after 900 s, 69 ± 30 kPa for Hydr3%, and 9 ± 4 kPa for Hydr2%. The relaxation modulus of Hydr2% at 45 °C almost equals the relaxation modulus at 4 °C, which indicates that almost all physical interactions are relaxed and the stress still present only originates from the covalently crosslinked network. This relaxation behavior was also observed in the frequency sweeps of the rheology measurements at 45 °C, however the longest timescale probed was still too short for all the reversible bonds to relax.

### 3.4. Cell Viability

**Figure 10:**
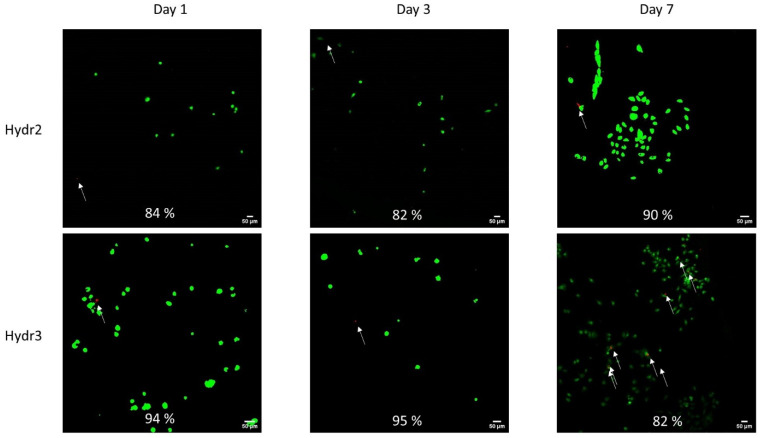
Cell viability assessed after 1, 3 and 7 days of incubation in cell culture medium at 37 °C and 5% CO_2_ humidified atmosphere. The living cells were visualized in green using Calcein AM (488/515) and the dead cells were visualized red using BOBO-3 Iodide (570/602) and indicated with white arrows.

The cell viability was assessed after 1, 3 and 7 days of incubation of chondrocytes inside the hydrogel network. Overall, high cell viability was observed over an incubation period of one week (Figure 10). Initially, a higher cell viability was observed for Hydr3% compared to Hydr2%. After 1 day, 94% of the chondrocyte were still alive in Hydr3% and 84% in Hydr2%. After 3 days, 95% of cells were alive in Hydr3% and 82% were alive in Hydr2%. After 7 days, 83% of cells were alive in Hydr3% and 90% in Hydr2%, showing a higher cell viability for Hydr2% over Hydr3%. However, all these differences are only minor and hence both hydrogels can be considered to have similar cyto-toxic behavior. It was also observed that much more chondrocytes were present after seven days of incubation, which indicates that the chondrocytes proliferated and multiplied over this time span. However, at each time point different samples were compared so further studies are required to confirm this hypothesis.

## 4. Conclusions

The produced thermo-responsive co-polymers, P(NIPAAm-co-Cys) and P(NiPAAM-co-HEAacr) spontaneously reacted in a thiol-ene reaction, yielding a covalently crosslinked hydrogel network with a limited number of polymers leaking out of the hydrogel. Increasing the temperature of these hydrogels above their transition temperature of 31 °C induced the formation of additional physical interactions increasing the hydrogel stiffness. These physical interactions are reversible and can relax over time, which drastically changed the material’s overall behavior from elastic to visco-elastic. Different co-polymers were synthesized with different incorporation ratios of functional monomers, ranging from 1% to 10%. Increasing the functional monomer ratios decreased the solubility of the co-polymer in cell culture medium and at an incorporation ratio above 5%, the polymers became insoluble. The most loosely crosslinked network was formed by the polymers with 1% functional monomer and it possessed the most outspoken thermo-responsive behavior, where a 60-fold increase in complex modulus was measured using rheology. The stiffest hydrogel was formed by mixing polymers with 3% functional co-monomer, where the incorporation ratio is high enough to produce a densely crosslinked network and the co-polymers still remain sufficiently soluble. At 4 °C, a compression modulus of 27 kPa was measured. Increasing the temperature to 45 °C (above the T_cp_ of 31 °C) increased the compression modulus eight times, to 226 kPa. The developed hydrogels, with optimized functional monomer ratio, reach stiffnesses more than ten times higher compared to the state of the art using similar polymers [36,37]. By selecting the right crosslink density, the hydrogel can match different stiffnesses of different types of tissue. Furthermore, good cell viability was confirmed by a LIVE/DEAD assay after 7 days of culturing chondrocytes inside the hydrogel. Altogether, a versatile platform was created to embed different cells to investigate the effect of the micro-environment on the phenotype of the embedded cells.

Different possibilities to further modify these materials exist. The straight-forward production technique of the hydrogel allows the application of different printing (e.g., bioprinting, electrospinning) and administration techniques (e.g., injectability) which can be exploited. Further advances or modifications in the synthesis of the polymers can introduce stronger bonds (e.g., more reactive functional groups, multiple reaction sites) or additional responsive behavior (e.g., light-responsive, enzyme responsiveness).

The micro-environment of cells plays a major role in different diseases in different types of tissue, as there are diseases related to load bearing tissue (e.g., osteoarthritis) as well as cancer related diseases (e.g., brain tumors) and many more. To this end, a hydrogel with reversible tunable viscoelastic properties in which cells can be seeded would revolutionize these mechanobiology studies as it would allow to conveniently modulate the 3D micro-environment of the cells in vitro, while leaving the cells in situ. In future work, chondrocytes will be embedded inside the hydrogel and mechanical loading conditions will be applied on these constructs while being incubated at different temperatures, thereby changing the nature of the hydrogel. Changes in the phenotype induced by mechanical loading on the cells in different micro-environments will give more insight in the role of the cell’s mechanical micro-environment and opens the possibility to optimize the tissue’s mechanical properties in order to cure diseases like osteoarthritis with adequate physical exercise.

## Figures and Tables

**Table 1:** Overview of the synthesized co-polymers with different feed ratios of functional monomer (HEAacr and Cys), ranging from 1% to 5%. Percentage following the name indicates the feed ratio of the corresponding functional monomer. Yield represents the overall yield of the polymer synthesis starting from the monomers and includes the post-polymerization modification. Incorporation ratio in the polymer chain was calculated using NMR and Ellman’s method for Cys. Molecular weight (Mw) and dispersity (Đ) were measured using SEC. * Spill during transfer to dialysis membrane. ** Solubility too low to obtain reliable results.

Name	Yield	HEA (NMR)	HEAacr (NMR)	Mw (×10^3^ g/mol)	Đ	Name	Yield	Cys (NMR)	Ellman’s Method	Mw (×10^3^ g/mol)	Đ
*HEAacr 1%*	44%	0.88%	0.67%	11.4	1.43	*Cys 1%*	46%	0.95%	0.74%	11.5	1.37
*HEAacr 2%*	43%	1.8%	1.8%	28.0	1.89	*Cys 2%*	65%	1.9%	1.8%	19.3	1.51
*HEAacr 3%*	46%	2.7%	2.8%	46.8	2.19	*Cys 3%*	49%	3.0%	2.6%	23.5	1.62
*HEAacr 4%*	77%	3.8%	3.3%	127 **	4.99 **	*Cys 4%*	29% *	3.8%	3.9%	18.0	1.58
*HEAacr 5%*	66%	**	**	**	**	*Cys 5%*	57%	4.7%	4.3%	28.3	1.55

## Data Availability

Data are contained within the article.

## Supplementary Materials

The following supporting information can be downloaded at: https://www.mdpi.com/article/10.3390/polym16091264/s1, Figure S1: NMR spectra of HEAacr; Table S1: Overview intensities measured in NMR spectra P(NiPAAm-co-HEAacr); Table S2: Obtained incorporation ratios from NMR spectra for P(NiPAAm-co-HEAacr); Figure S2: NMR spectra of Cys3.; Table S3: Overview intensities measured in NMR spectra P(NiPAAm-co-Cys); Table S4: Obtained incorporation ratios from NMR spectra for P(NiPAAm-co-Cys); Table S5: Obtained incorporation ratios from Ellman’s method for P(NiPAAm-co-Cys); Figure S3: FITR of Cys with different incorporation ratio and annotated peaks; Figure S4: FITR of HEAacr with different incorporation ratio and annotated peaks; Table S6: numerical values of Figure 4, mechanical properties of hydrogels consisting of polymers with a different incorporation ratio determined by rheology; Figure S5: Frequency and strain sweep for Hydr2% at 4°C and 45°C; Table S7: numerical values of Figure 9, mechanical properties of hydrogels consisting of polymers with a different incorporation ratio determined by rheology; Figure S6: Compression-relaxation test of hydr2% and hydr3% at 4 °C and 45 °C; Movie S1.
