# Supplementary material for: A Spontaneous In Situ Thiol-Ene Crosslinking Hydrogel with Thermo-Responsive Mechanical Properties
Source: Polymers (Basel). 2024 May 1;16(9):1264. doi: 10.3390/polym16091264 (PMC11085619; doi:10.3390/polym16091264)
Supplement: Supplementary file 1 [file polymers-16-01264-s001.zip › polymers-2971081-supplementary.pdf]

Supporting information:

A Spontaneous In Situ Thiol-Ene Crosslinking Hydrogel with Thermo-Responsive Mechanical Properties

Andreas Aerts [1], Maxim Vovchenko [2,3], Seyed Ali Elahi [2,4], Rocío Castro Viñuelas [4,5], Tess De Maeseneer [6], Martin Purino [7], Richard Hoogenboom [7], Hans Van Oosterwyck [2,8], Ilse Jonkers [4], Ruth Cardinaels [6] and Mario Smet [1,*]

[1] Laboratory of Organic Material Synthesis, Polymer Chemistry and Materials, Department of Chemistry, KU Leuven, Celestijnenlaan 200f, P.O. Box 2404, 3001 Leuven, Belgium; andreas.aerts@kuleuven.be (A.A.)
[2] Biomechanics Section, Department of Mechanical Engineering, KU Leuven, Celestijnenlaan 300C, P.O. Box 2419, 3001 Leuven, Belgium.
[3] Laboratory for Soft Matter and Biophysics, Department of Physics and Astronomy, KU Leuven, Celestijnenlaan 200D, P.O. Box 2416, 3001 Leuven, Belgium
[4] Human Movement Biomechanics Research Group, Department of Movement Sciences, KU Leuven Tervuursevest 101, P.O. Box 1501, 3001 Leuven, Belgium.
[5] Laboratory for Tissue Homeostasis and Disease, Department of Development and Regeneration, KU Leuven, Herestraat 49, P.O. Box 813, 3000 Leuven, Belgium
[6] Rheology and Technology, Soft Matter, Department of Chemical Engineering, KU Leuven, Celestijnenlaan 200J, P.O. Box 2424, 3001 Leuven, Belgium;
[7] Supramolecular Chemistry Group, Department of Organic and Macromolecular Chemistry, UGent, Krijgslaan 281, Building S4, 9000 Ghent, Belgium;
[8] Prometheus, Division of Skeletal Tissue Engineering, KU Leuven, Herestraat 49, P.O. Box 813, 3000 Leuven, Belgium
* Correspondence: mario.smet@kuleuven.be

^{1}HNMR spectroscopy

P(NIPAAM-CO-HEAACR)

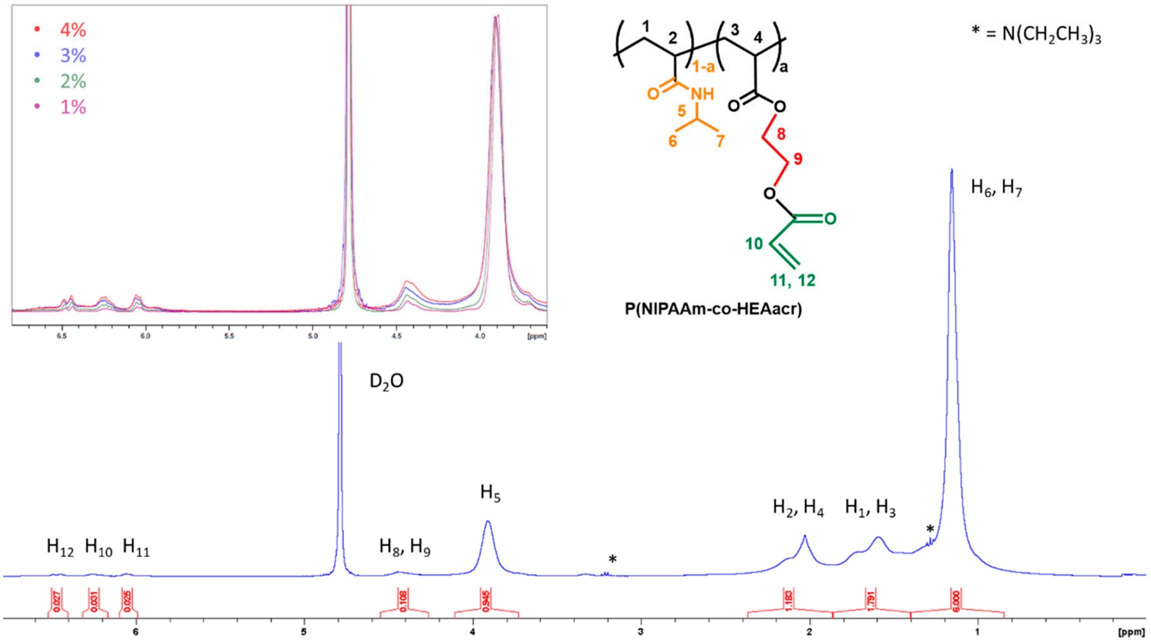

Fig. S1: Spectra of HEAacr: inset represents different incorporation ratio. Normalized to peak 3.91 ppm.

DIFFERENT RATIOS HEAACR

$\delta(ppm)$	*Proton*	*# protons*	*Intensity HEAacr1*	*Intensity HEAacr2*	*Intensity HEAacr3*	*Intensity HEAacr4*
6.46	H_{12}	1	0.007	0.019	0.027	0.030
6.24	H_{10}	1	0.006	0.019	0.031	0.039
6.05	H_{11}	1	0.007	0.017	0.025	0.030
4.79	D_2O	/	/	/	/	/

4.40	H_8, H_9	4	0.035	0.072	0.108	0.154
3.91	H_5	1	1.000	1.000	1.000	1.000
2.12	H_2, H_4	2	1.108	1.137	1.183	1.397
1.63	H_1, H_3	4	1.864	1.814	1.791	1.800
1.13	H_6, H_7	6	5.922	5.82	5.682	5.688

Table S1: Overview intensities measured in NMR spectra P(NiPAAm-co-HEAacr)

$$\frac{HEA}{NIPAAm} = \frac{I(\mathrm{H_8}, \mathrm{H_9})}{4} \quad e.g.(HEAacr3) = \frac{0.108}{4} = 2.7\ \%$$

$$\frac{HEAacr}{NIPAAm} = \frac{I(\mathrm{H_{10}}, \mathrm{H_{11}}, \mathrm{H_{12}})}{4}$$

$$e.g.(HEAacr3) = \frac{0.025 + 0.031 + 0.027}{3} = 2.7\ \%$$

Name	*Feed*	*Measured HEA (NMR)*	*Measured HEAacr (NMR)*
HEAacr 1%	1 %	0.88 %	0.67 %
HEAacr 2%	2 %	1.8 %	1.8 %
HEAacr 3%	3 %	2.7 %	2.7 %
HEAacr 4%	4 %	3.8 %	3.3 %

Table S2: Obtained incorporation ratios from NMR spectra for P(NiPAAm-co-HEAacr)

P(NiPAAm-co-cys)

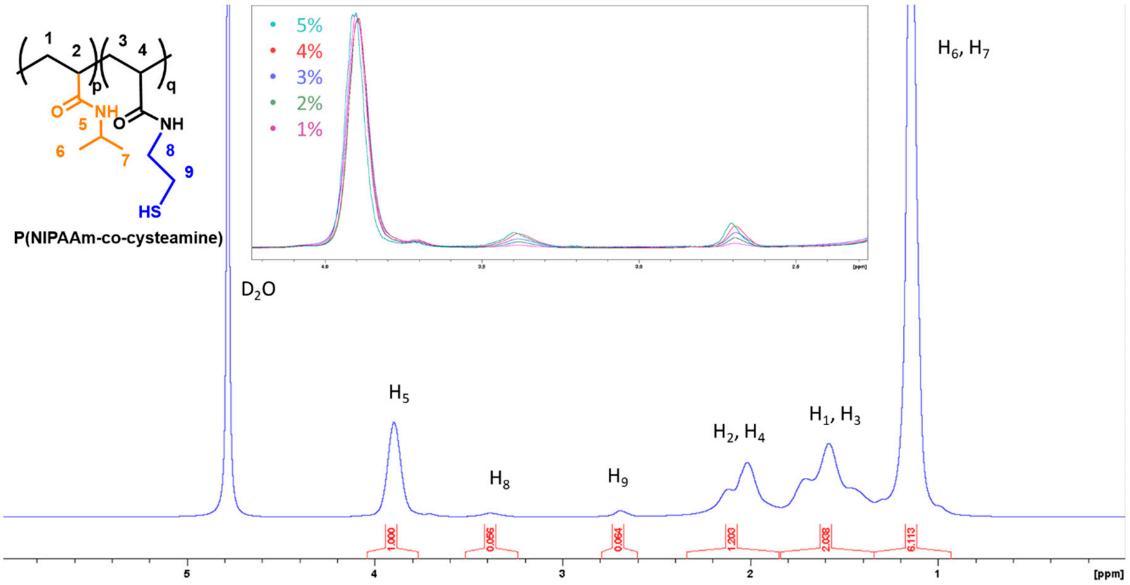

Fig.S2: Spectra of Cys3; inset represents different incorporation ratio. Normalized to peak 3.91 ppm.

$\delta(ppm)$	*Proton*	*# protons*	*Intensity Cys 1*	*Intensity Cys 2*	*Intensity Cys 3*	*Intensity Cys 4*	*Intensity Cys 5*
4.79	D_2O		/	/	/	/	/
3.90	H_5	1	1.000	1.000	1.000	1.000	1.000
3.39	H_8	2	0.017	0.035	0.056	0.076	0.095
2.69	H_9	2	0.021	0.039	0.064	0.078	0.091
2.10	H_2, H_4	2	1.131	1.193	1.203	1.235	1.126
1.60	H_1, H_3	4	1.909	1.875	2.038	1.880	1.977
1.14	H_6, H_7	6	6.024	6.091	5.988	6.192	6.000

Table S3: Overview intensities measured in NMR spectra P(NiPAAm-co-Cys)

$$\frac{Cys}{NIPAAm} = \frac{I(\mathrm{H}_8, \mathrm{H}_9)}{4} \quad e.g.(Cys3) = \frac{0.056 + 0.064}{4} = 3.0\ \%$$

Name	*Feed*	*Measured (NMR)*
Cys 1%	1 %	0.95 %
Cys 2%	2 %	1.85 %
Cys 3%	3 %	3.00 %
Cys 4%	4 %	3.85 %
Cys 5%	5%	4.65 %

Table S4: Obtained incorporation ratios from NMR spectra for P(NiPAAm-co-Cys)

ELLMAN'S METHOD

Name	*Concentration solution (mmol/L)*	*Absorbance (A)*	*Percentage free thiols*	*Conversion*
**CYS1**	7.35	0.774	0.74%	74%
**CYS2**	2.77	0.690	1.76%	88%
**CYS3**	7.33	2.74	2.64%	88%
**CYS4**	3.41	1.88	3.89%	97%
**CYS5**	7.31	4.36	4.22%	84%

Table S5: : Obtained incorporation ratios from Ellman's method for P(NiPAAm-co-Cys)

$$molar\ absorption\ coefficient\ (\varepsilon) = 14\ 150\ M^{-1}cm^{-1}$$

$$Percentage\ free\ thiols\ = \frac{\frac{Absorbance\ (A)}{molar\ absorption\ coefficient\ (\varepsilon)}}{concentration\ solution}$$

$$Conversion = \frac{Percentage\ free\ thiols}{feed\ ratio}$$

FTIR SPECTROSCOPY

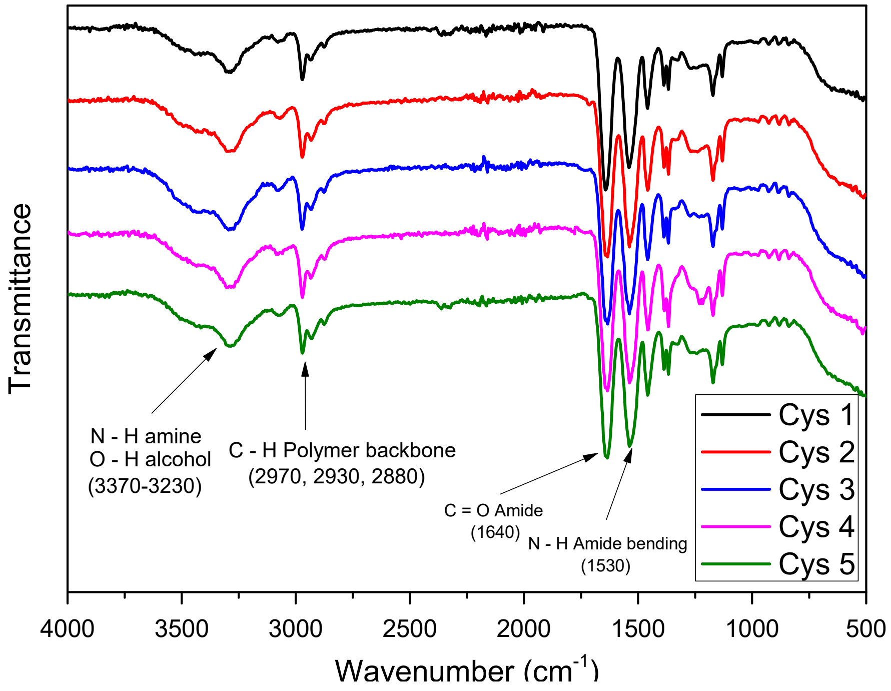

Fig.S3: FITR of Cys with different incorporation ratio and annotated peaks.

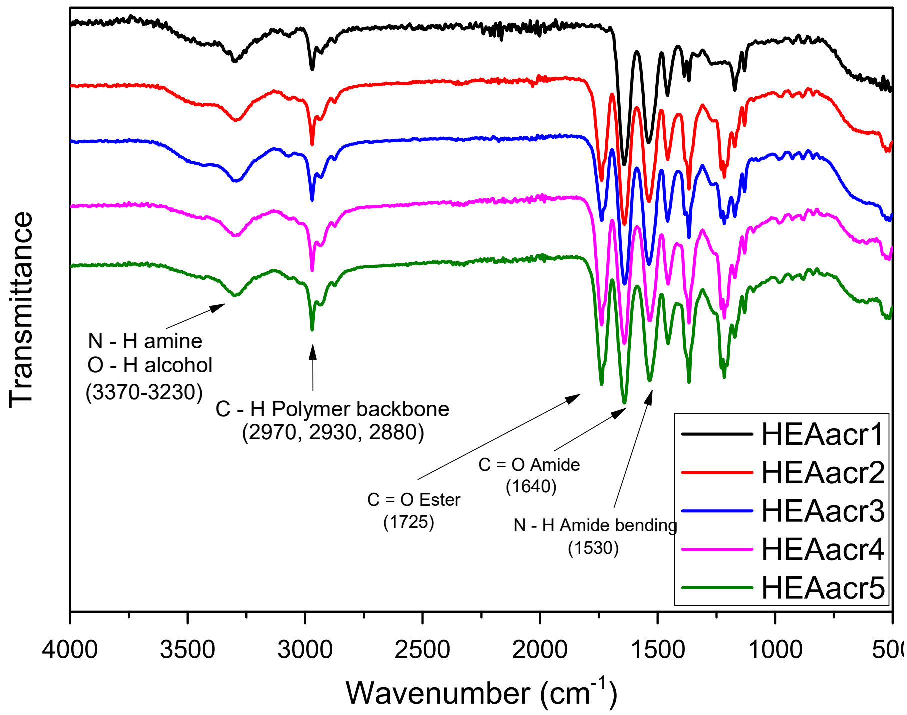

Fig.S4: FITR of HEAacr with different incorporation ratio and annotated peaks.

	Cold 4 °C				*Hot 45 °C*			
Name	**Storage modulus G' (kPa)**	**Loss modulus G'' (kPa)**	**Complex modulus G* (kPa)**	**Loss factor tan(δ)**	**Storage modulus G' (kPa)**	**Loss modulus G'' (kPa)**	**Complex modulus G* (kPa)**	**Loss factor tan(δ)**
Hydr 1%	**0.33 ± 0.08**	**0.056 ± 0.002**	**0.34 ± 0.08**	**0.17 ± 0.04**	**10.1 ± 1.6**	**14.7 ± 2.8**	**18.8 ± 2.9**	**1.45 ± 0.23**
Hydr 2%	**6.55 ± 0.59**	**0.442 ± 0.163**	**6.56 ± 0.59**	**0.068 ± 0.03**	***29.7 ± 5.5***	**34.7 ± 6.6**	**45.7 ± 8.6**	**1.17 ± 0.01**
Hydr 3%	**11.0 ± 2.7**	**0.269 ± 0.129**	**11.0 ± 2.7**	**0.025 ± 0.01**	***44.3 ± 11.9***	**31.5 ± 9.9**	**54.4 ± 15.3**	**0.71 ± 0.07**
Hydr 4%	**3.08 ± 0.49**	**0.155 ± 0.054**	**3.08 ± 0.49**	**0.05 ± 0.01**	***14.4 ± 1.4***	**12.3 ± 1.3**	**18.9 ± 1.9**	**0.86 ± 0.03**

Table S6: Numerical values of Figure 4, mechanical properties of hydrogels consisting of polymers with a different incorporation ratio determined by rheology

Frequency and strain sweep Hydr2%

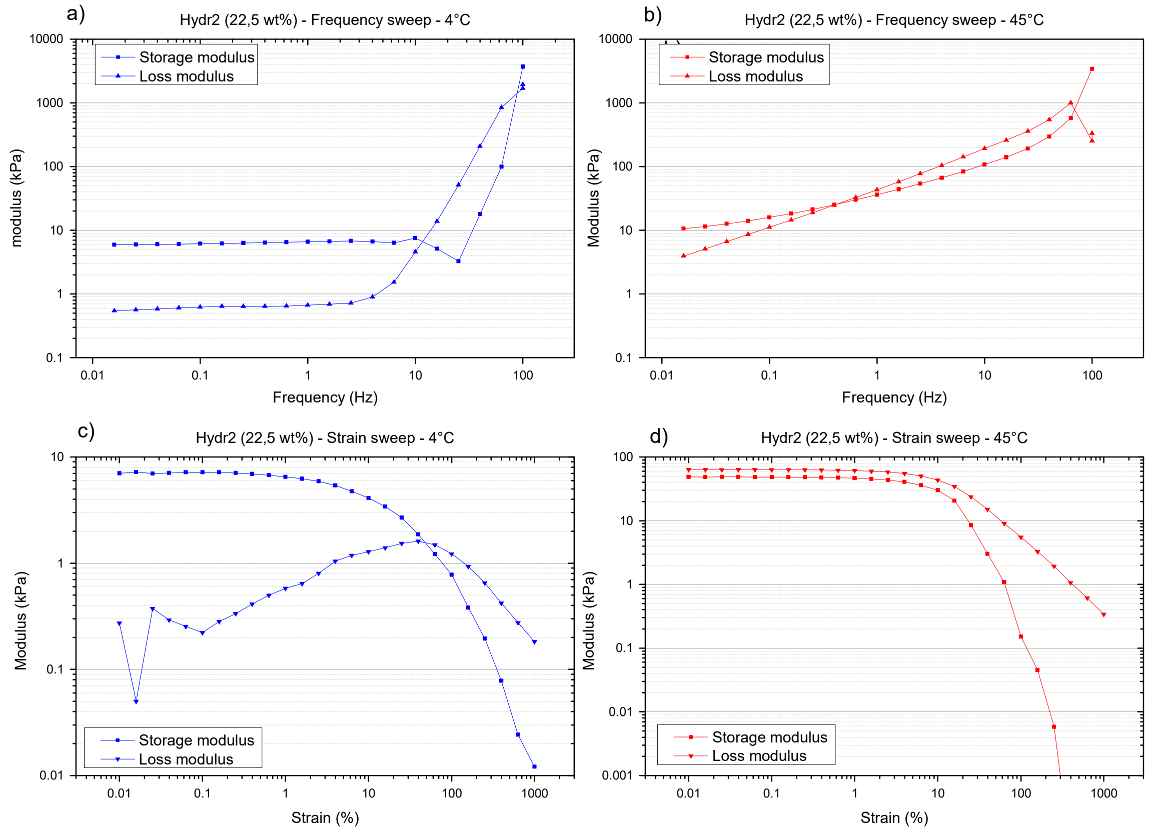

Fig.S5: Frequency and strain sweep for Hydr2% at 4°C and 45°C; Frequency and strain sweep for Hydr2% at 4°C (blue) and 45°C (red), a) frequency sweep at 4°C, b) frequency sweep at 45 °C, c) strain sweep at 4 °C, d) strain sweep at 45 °C

	Cold 4 °C		Hot 45 °C	
Name	**Young's modulus (kPa)**	**Relaxation modulus (kPa)**	**Young's modulus (kPa)**	**Relaxation modulus (kPa)**
Hydr 2%	**9.86 ± 1.56**	**5.98 ± 1.41**	***94.5*** **± 30.6**	**6.12 ± 1.14**
Hydr 3%	**21.7 ± 4.3**	**12.6 ± 3.4**	***181.1 ± 31.7***	**55.4 ± 26.1**

Table S7: Numerical values of Figure 9, mechanical properties of hydrogels consisting of polymers with a different incorporation ratio determined by rheology;

Compression – relaxation: data

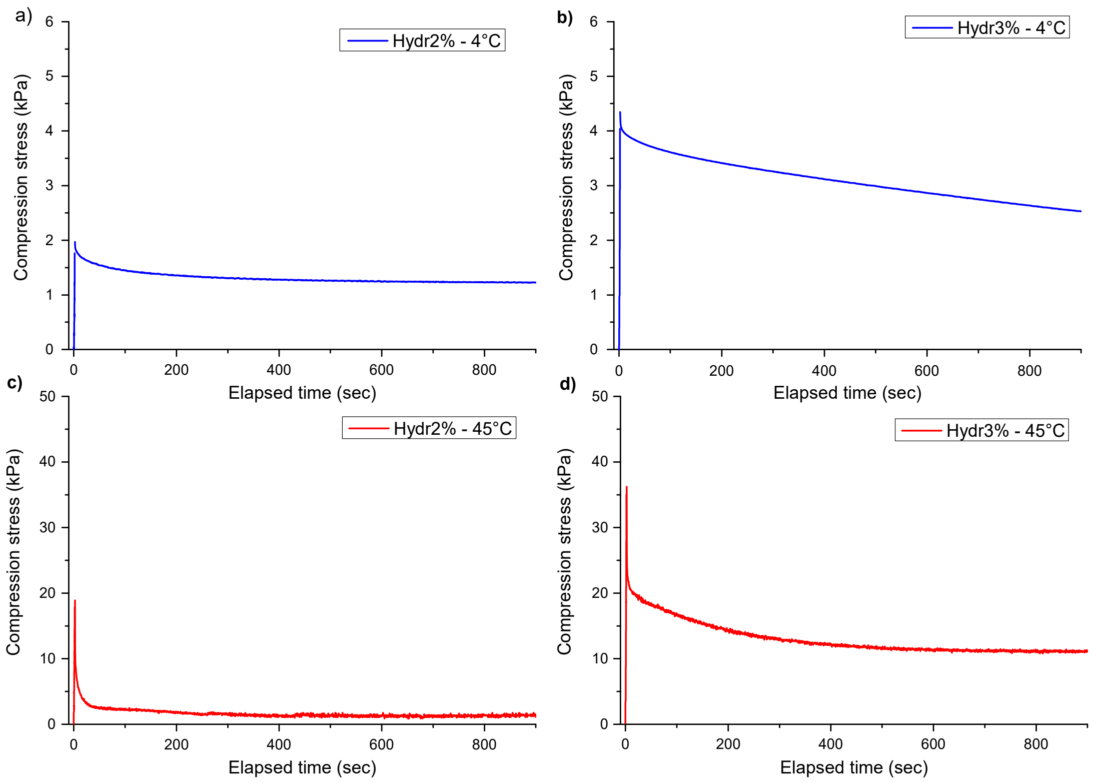

Fig.S6: Compression-relaxation test of hydr2% and hydr3% at 4 °C and 45 °C; a) hydr2% at 4 °C, b) hydr3% at 4 °C, c) hydr2% at 45 °C, d) hydr3% at 45 °C
